# Systematic population spike delays across cortical layers within and between primary sensory areas

**DOI:** 10.1038/s41598-017-15611-2

**Published:** 2017-11-10

**Authors:** Gijs Plomp, Christoph M. Michel, Charles Quairiaux

**Affiliations:** 1 Perceptual Networks Group, Dept. of Psychology, University of Fribourg, Fribourg, Switzerland; 2 Functional Brain Mapping Laboratory, Dept. of Fundamental Neuroscience, University of Geneva, Geneva, Switzerland

## Abstract

The coordinated propagation of activity across cortical layers enables simultaneous local computation and inter-areal interactions. A pattern of upward propagation from deeper to more superficial layers, which has been repeatedly demonstrated in spontaneous activity, would allow these functions to occur in parallel. But it remains unclear whether upward propagation also occurs for stimulus evoked activity, and how it relates to activity in other cortical areas. Here we used a new method to analyze relative delays between spikes obtained from simultaneous laminar recordings in primary sensory cortex (S1) of both hemispheres. The results identified systematic spike delays across cortical layers that showed a general upward propagation of activity in evoked and spontaneous activity. Systematic spike delays were also observed between hemispheres. After spikes in one S1 the delays in the other S1 were shortest at infragranular layers and increased in the upward direction. Model comparisons furthermore showed that upward propagation was better explained as a step-wise progression over cortical layers than as a traveling wave. The results are in line with the notion that upward propagation functionally integrates activity into local processing at superficial layers, while efficiently allowing for simultaneous inter-areal interactions.

## Introduction

The structural organization of cerebral cortex consists of six cortical layers that can be distinguished based on cell-types, circuits and functional selectivity of activity^1–4^. Coordinated activity across cortical layers critically underlies both local processing within cortical areas, and directed interactions with other cortical and sub-cortical areas. The circuitry of interlaminar structural connections is highly complex but a simplified, canonical circuit of excitatory axonal projections and activations has been proposed^2,5^. In this canonical pattern layer IV (L4) is the main recipient of thalamic sensory inputs that relays stimulus-evoked activity to supragranular layers (L2/3), which in turn relay information to infragranular layers (L5, L6). This is in line with activation patterns observed in primary sensory regions after stimulation^4,6^.

Recently however, an alternative model of evoked activity has been proposed in which the thalamus directly sends copies of sensory information to both L4 and infragranular layers^7^. It has been shown that many L5 cells respond to sensory stimulation before L2/3 neurons do, suggesting a more important role for infragranular layers in stimulus-evoked activity. Such an account of evoked activity bears resemblance to established accounts of spontaneous activity. Spontaneous activity typically propagates upward from infra- to supragranular layers as shown *in vivo* ^6,8–11^, while *in vitro*, L5 activity has been shown to be necessary for sustained activity at supragranular layers^12^. Upward propagation in stimulus-evoked activity has so far only been shown using functional connectivity analysis of local field potentials (LFPs)^13^, and whether this holds on the level of spiking activity remains unclear.

Here, we analyzed relative delays between spikes at each cortical layer of S1 (barrel cortex), in both hemispheres of rats for activity evoked by unilateral whisker stimulation and for spontaneous activity. In the case of predominant upward propagation a systematic pattern of delays would be expected such that after a spike in a given layer, subsequent spikes in the other layers show increased delays in the upward direction (toward supragranular layers) but not in the downward direction (toward infragranular layers). Additionally, we predicted that relative to spiking activity in one S1, the subsequent spikes in the other S1 would first occur in deeper layers (L4, L5, L6), because functional connectivity results previously showed that interactions between the S1s specifically target L4 and infragranular layers, in line with known axonal and functional connections^13–17^.

The results showed a laminar pattern of systematic delays that confirm a predominant upward propagation of activity both in stimulus-evoked and in spontaneous activity. The spike-to-spike delays in S1 of the other hemisphere were shortest for infragranular layers, in line with a specific targeting of deeper layers in S1-S1 interactions. Further analyses showed that upward propagation was better characterized as a step-wise propagation across cortical layers than as a wavelike propagation across tissue.

## Results

### Population spiking activity

**Figure 1:**
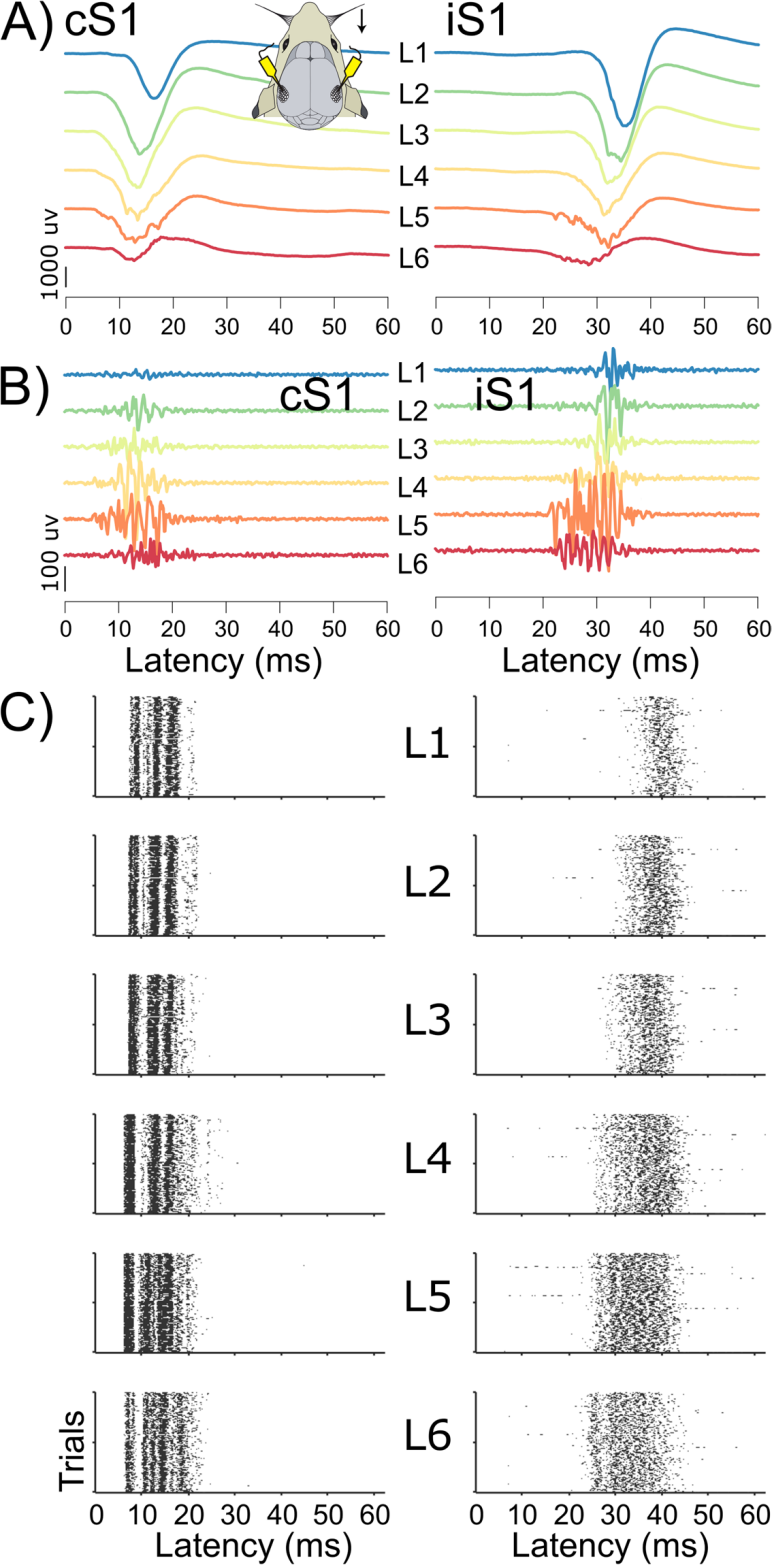
LFP and spiking activity in each S1 after unilateral whisker stimulation. (**A**) Broadband evoked LFP signals at each cortical layer of S1 contralateral (cS1) and ipsilateral (iS1) to stimulation, on a single trial (**B**) Corresponding MUA activity (**C**) Illustrative raster plots of evoked population spiking activity for 100 repeated whisker stimulations in one animal.

**Figure 2:**
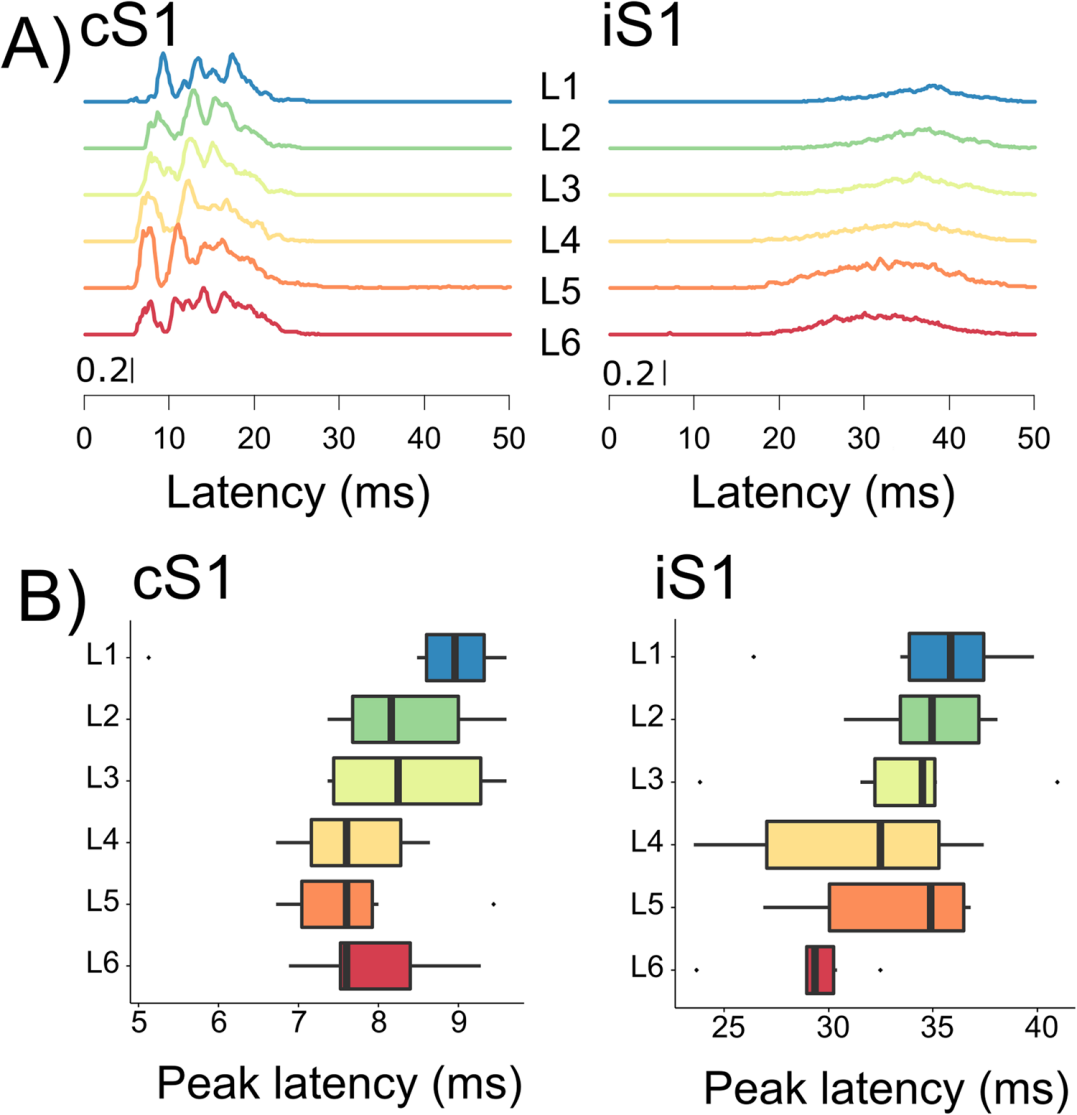
Average evoked population activity in the two S1s. (**A**) Average spike likelihood (probability) across animals for each layer in contra- and ipsilateral S1. (**B**) Boxplots of peak latency distributions for spike likelihoods in cS1 shortly after stimulation (<10 ms) and ipsilateral S1.

We used stimulus-evoked and spontaneous data recorded during isoflurane anesthesia with laminar probes positioned at each cortical layer in the course of a whisker stimulation paradigm^13,18,19^. Figure 1 illustrates a single sweep of stimulus-evoked LFPs and the corresponding multi-unit activity (MUA; bandpassed 750–2000 Hz) in S1 contralateral (cS1) and ipsilateral (iS1) to unilateral whisker stimulation. Through adaptive thresholding of the MUA we determined population spiking activity (see also Methods)^20^. The resulting single trial population spiking activities for 100 whisker stimulations are illustrated in Fig. 1C. From individual evoked population activity we calculated the spike likelihood across trials; Fig. 2 shows the grand-average evoked spike likelihood. In line with previous work^4,6^, the distributions of initial peak latencies in cS1 (Fig. 2B) suggest fast activation of all cortical layers, with slightly earlier responses in L4, L5 as compared to supragranular layers (although median L6 latencies appeared indistinguishable). Peak latencies of population spike likelihoods in iS1 occurred at longer latencies, and although they showed considerable variation these suggest that spiking activity first peaked in L6 and L4, followed by activity in supragranular layers (Fig. 2B, supplementary Table S1 for average peak latencies and 95% confidence intervals). The pattern of evoked activity in iS1 resembles the propagation of activity typically seen in spontaneous activity: abundant spiking in infragranular layers, followed by sparser activity in supragranular layers^6^ (c.f. Fig. 1C). This pattern obtained from population spiking activity resembles the results from LFP and MUA analysis of these data^13^.

### Upward propagation in evoked and spontaneous activity

**Figure 3:**
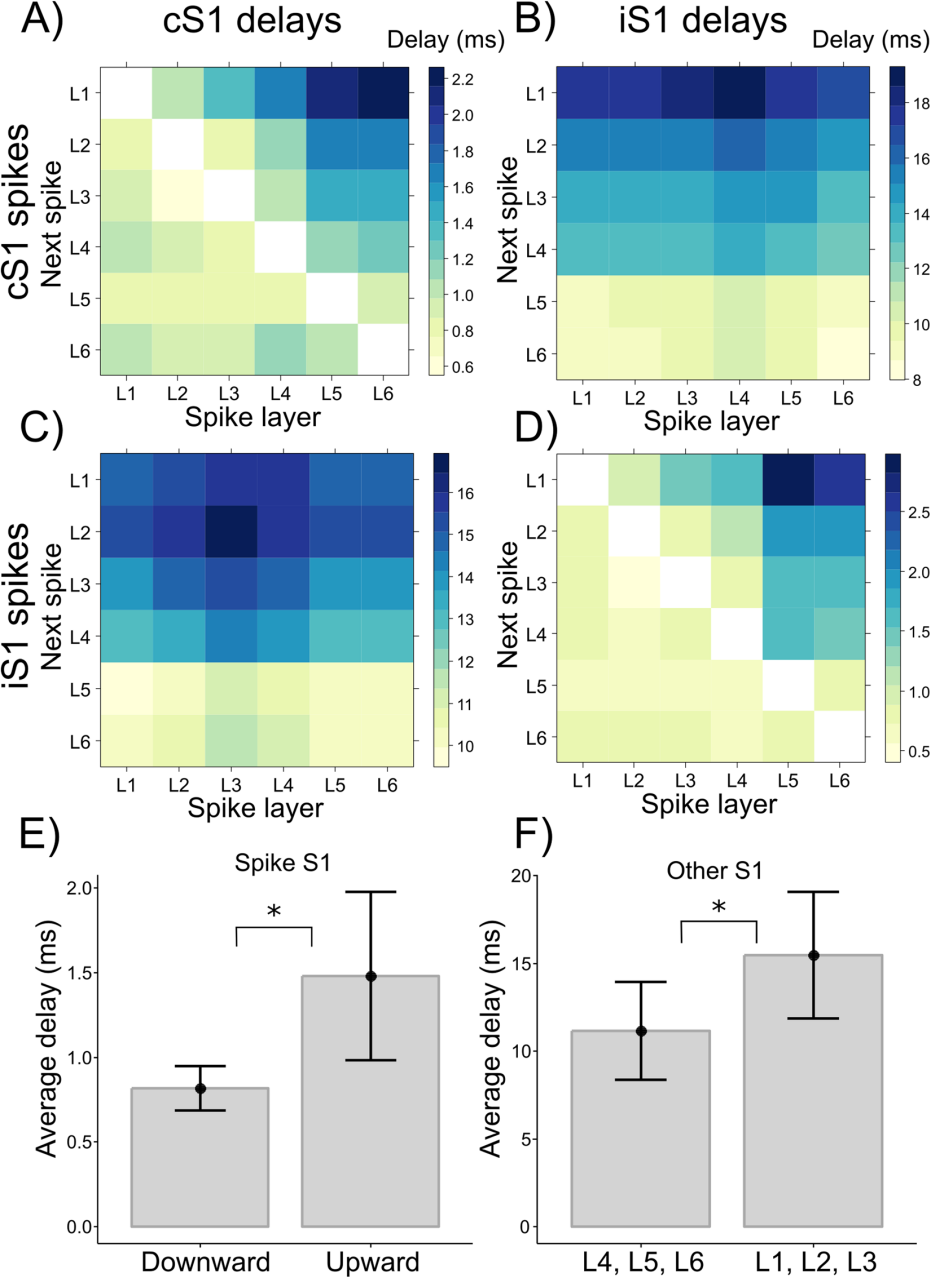
Evoked spike-to-spike delays. The two top panels (A,B) show relative delays after spikes in cS1 to stimulation. The spike layer is on the abscissa, delays for the next spike are plotted for each cortical layer on the ordinate, for the subsequent spikes in cS1 (**A**) and iS1 (**B**). Panels C and D depict in the same way the spike delays in cS1 (**C**) and iS1 (**D**) following spikes in iS1. (**E**) shows average upward and downward delays within the spike S1, while F) shows average delays in deep (L4, L5, L6) and superficial (L1, L2, L3) layers of the other S1. Error bars denote 95% confidence intervals across animals (n = 6).

To determine how population spiking activity propagates across cortical layers, we calculated for each spike the relative delay with which the next spike occurred in the other cortical layers (spike-to-spike delay) of the S1 where the spike occurred (spike S1), and in the S1 of the other hemisphere (other S1). We calculated spike-to-spike delays separately for spikes occurring in cS1 and iS1. For evoked activity the average delays after spikes in cS1 are shown in Fig. 3A,B, for each layer of cS1 and iS1 respectively; the delays after spikes in iS1 are shown in Fig. 3C,D for subsequent spikes in cS1 and iS1 respectively.

Following spikes in cS1, spike-to-spike delays in the other cS1 layers systematically increased in the upward direction: after a spike in L5, for example, it took longer for the next spike to occur in L3 than in L4, and again longer for one to occur in L2 or L1 (Fig. 3A). Spike-to-spike delays increased in the upward direction following spikes in each cortical layer. In the downward direction, delays did not increase with cortical layer, indicating that stimulus-evoked activity in cS1 in general propagates upward across cortical layers.

Following spikes in cS1, furthermore, the delays in iS1 also showed a laminar pattern consistent with upward propagation of population activity (Fig. 3B). For spikes in each layer of cS1, the shortest iS1 delays occurred in infragranular layers (L6, 9 ms on average) and delays progressively increased in the upward direction with each cortical layer. The longest delays were observed in supragranular layer L1 (17 ms, on average), showing an upward propagation of activity in the S1 ipsilateral to stimulation.

Following spikes in iS1, the delays in iS1 also showed a pattern consistent with upward propagation of activity. After iS1 spikes, delays for the next spikes increased in the upward direction, while for the downward direction delays did not systematically increase with layer (Fig. 3D). Furthermore, after iS1 spikes the laminar delays in cS1 also resembled an upward propagation of activity (Fig. 3C). Again the smallest delays occurred in infragranular layers, and increasingly longer delays occurred in the higher layers. Note that these delays were calculated on fewer spikes given the low activity in cS1 at longer latencies, and that cS1 spikes at longer latencies may result from feedback from multiple brain areas^21,22^. Nonetheless, the results support the interpretation of a pattern of predominant upward propagation of activity in stimulus evoked activity that is time-locked to activity in the other S1.

To test whether upward delays were longer than downward delays, and whether delays in the other S1 were shorter at deeper than at superficial layers we used non-parametric bootstrapping statistics (Methods). Given that delays following spikes in cS1 or iS1 were highly similar, we collapsed their data to quantify differences between delays in the upward and downward direction. In the spike S1, there was a statistically significant difference between average upward delays (right part above diagonal of Fig. 3A,D) and average downward delays (left part below diagonal of Fig. 3A,D), mean difference (M) = 0.66, bootstrapped 95% confidence interval (CI) [0.43–1.17], showing larger upward (1.48 +/− 0.47 ms) than downward delays (0.82 +/− 0.12 ms; Fig. 3E). This confirms that spike delays in the upward direction were prolonged while there was no systematic delay in the downward direction, and supports the hypothesis that activity predominantly propagates upward. Concerning the delays between the two S1s, we found a statistically significant difference between delays in deep layers (L4, L5, L6; lower halves of Fig. 3B,C) and delays in superficial layers (L1, L2, L3, upper halves of Fig. 3B,C) of the other S1, showing shorter interhemispheric delays in deep layers (11.15 +/− 2.66 ms) than in superficial layers (15.46 +/− 3.44 ms; Fig. 3F), M = 4.32, 95% CI [2.95–6.28].

**Figure 4:**
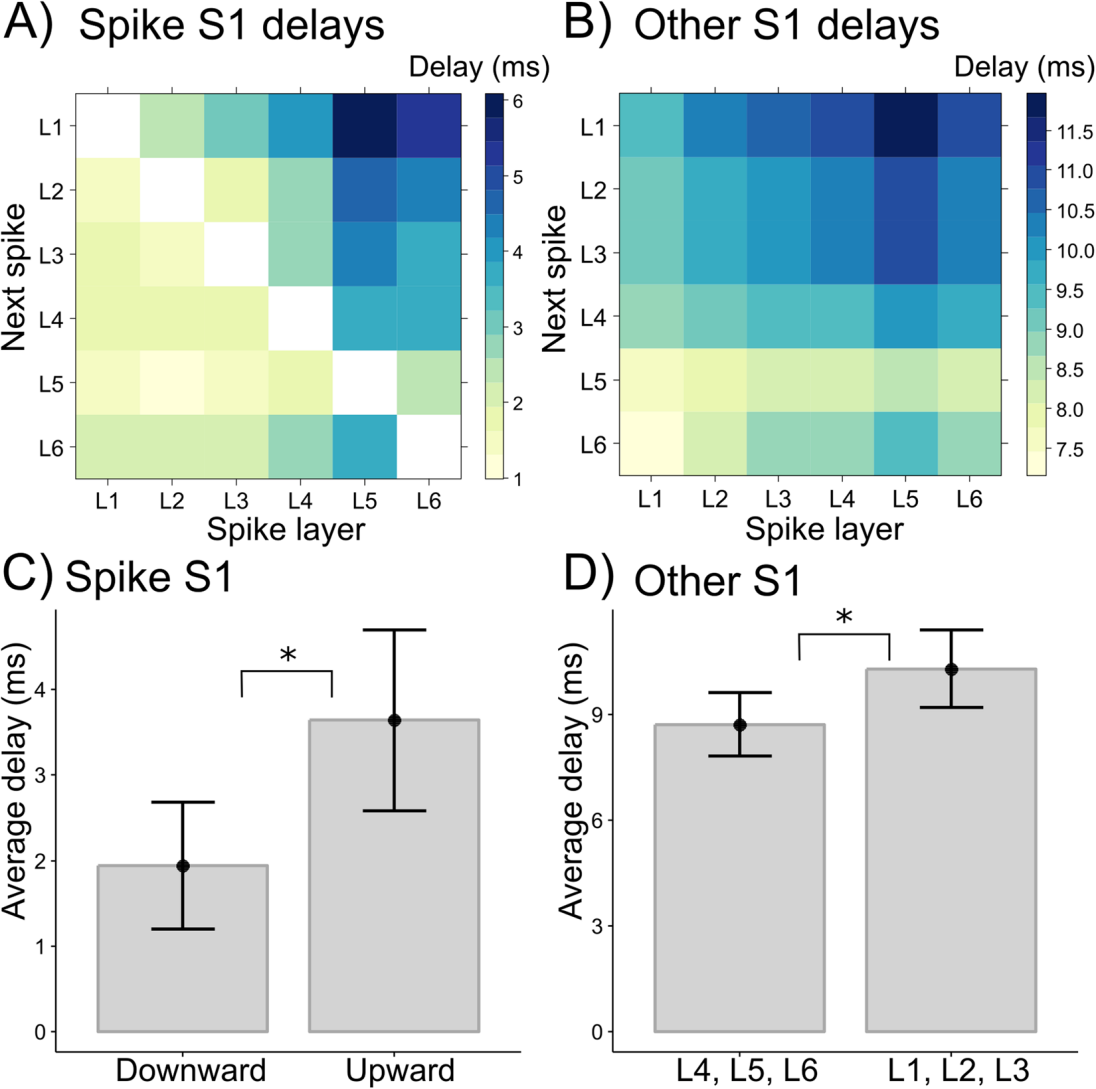
Spike-to-spike delays in spontaneous activity. (**A**) and (**B**) show the pattern of laminar delays in the spike S1 and the other S1, respectively. (**C**) shows average upward and downward delays within the spike S1; (**D**) shows average delays in the other S1 for deep (L4, L5, L6) and superficial layers (L1, L2, L3). Error bars denote 95% confidence intervals across animals.

We next investigated whether the stimulus evoked pattern of spike-to-spike delays also occurs in spontaneous activity. We selected 7 second epochs from the quiet inter-stimulus interval, at least 2 seconds after stimulation. Like for evoked activity, there was a statistically significant difference between upward delays (Fig. 4A, upper triangle) and downward delays (Fig. 4A, lower triangle), M = 1.7, 95% CI [1.36–2.08], with larger upward (3.64 +/− 1.01 ms) than downward delays (1.94 +/− 0.71 ms), in line with a predominant upward propagation of activity (Fig. 4C). Also similar to evoked activity, a systematic pattern of delays was seen in the other hemisphere, with shorter delays in the deep (8.72 +/− 0.86 ms) than in the superficial layers (10.30 +/− 1.05 ms; M = 1.58, 95% CI [0.91–2.07] (Fig. 4B,D).

While the spike-to-spike delay patterns for evoked and spontaneous activity were highly similar, the results showed differences in the average delay durations. Within the spike S1, delays were longer for spontaneous activity M = 1.37, 95% CI [0.96–1.89] with mean delay 2.3 +/− 0.7 vs. 1.0 +/− 0.2 ms, while in the other S1 delays were longer for evoked activity (13.3 +/− 2.9 vs. 9.5 +/− 0.9 ms), M = 3.79, 95% CI [2.14–6.54]. This suggests that evoked activity propagates upward at higher velocity, but has a slower relay to the other hemisphere.

### Control for spike rates

**Figure 5:**
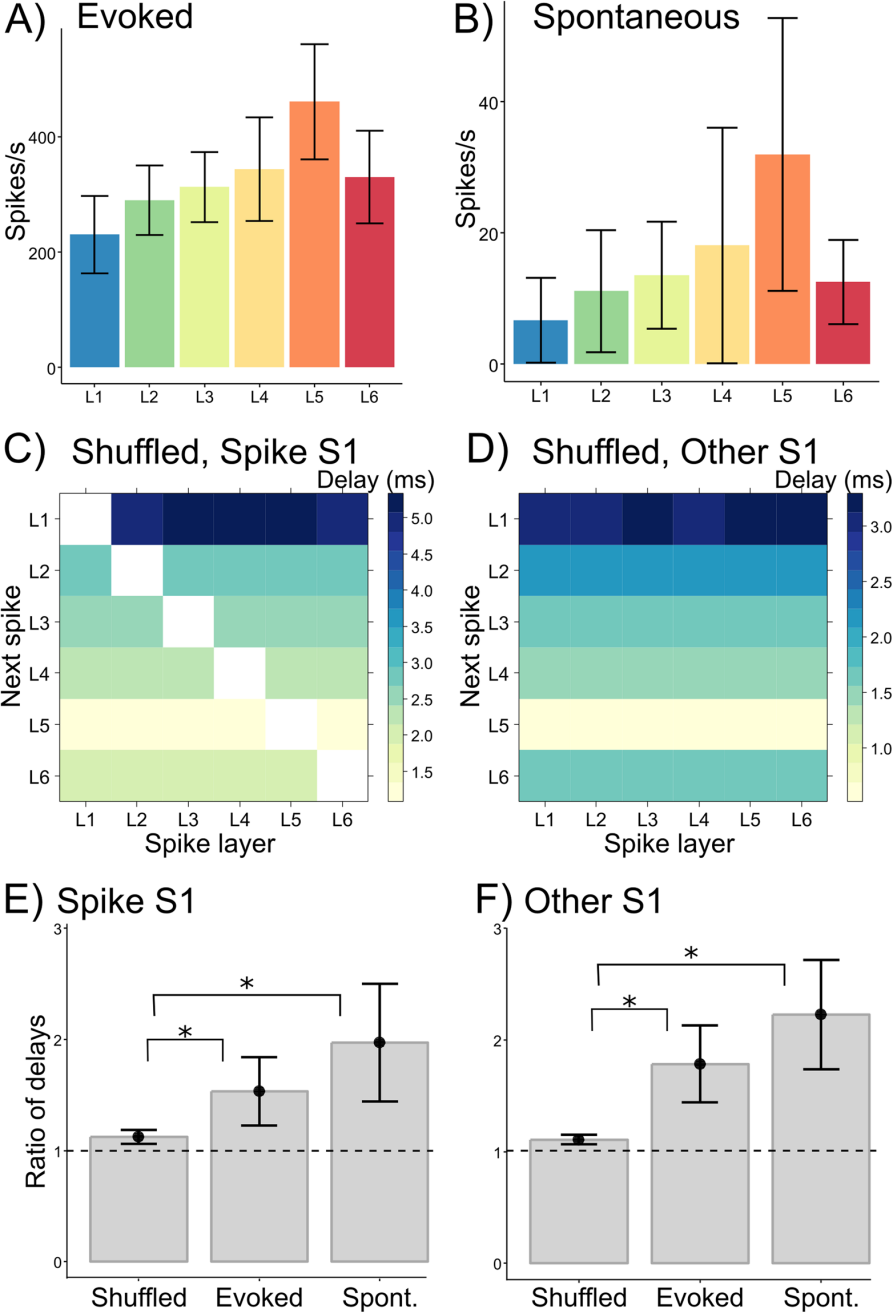
Spike frequency effects. (**A**) and (**B**) Show average spike rates per layer for evoked and spontaneous activity. Spike delays for data with shuffled spike labels (L1-L6) are depicted in (**C**) for the S1 where the spike occurred and (**D**) for the other S1. (**E**) and (**F**) Show the ratio of average delays after spikes in deep layers (L4-6) and superficial layers (after L1-3) for shuffled, evoked and spontaneous activity. Error bars denote 95% confidence intervals.

The results in both S1s were in line with a predominant upward propagation of activity across layers for evoked and spontaneous activity. In addition, they showed that spikes in one hemisphere were systematically delayed with respect to spikes in the other hemisphere, with increased delays from deeper to more superficial layers. A potential confound in these findings is that cortical layers typically show differences in spike rates. This can be problematic because higher spike rates lead to shorter inter-spike delays. We therefore investigated whether the observed laminar delays could be attributed to differences in spike rates. Figure 5A,B show spike rates in evoked and spontaneous activity. A linear mixed effects model with animals as a random factor showed that spike rates were non-uniform across layers in evoked activity *F*(5,25) = 20.3, *p* < 0.0001, nor in spontaneous activity, *F*(5,25) = 8.7, *p* < 0.001.

To test whether laminar differences in spike rates can account for the observed pattern of spike delays we randomly shuffled the labels (L1-L6) across all observed spikes (n = 1000 reshufflings per animal). This shuffling preserves the total number of spikes per layer but randomizes their temporal order. The spike-to-spike delays for shuffled data showed uniform delays for each layer (Fig. 5C,D). As expected, spike delays were shortest at L5, which showed the highest spike rate, and longest for L1, which showed the lowest spike rate. The shuffled delay matrix showed a similar delay pattern in the left part below the diagonal, as in the right part above the diagonal, reflecting laminar spike rates (Fig. 5C).

The spike delay pattern in shuffled data differed from that of real data in two important ways. The first difference was that shuffled data showed uniform delays for each layer, irrespective of where the spike occurred. For example, in shuffled data the next spike in L1 had the same delay after a spike in L2 as after a spike in L6, whereas in real data the delay for the next spike in L1 was longer after a spike in L6 than after a spike in L2 (Fig. 3A,D). To quantify this difference between shuffled and real data we calculated the ratio of average delays across layers after spikes in deep (L4, L5, L6) and superficial layers (L1, L2, L3). This ratio exceeds 1 when delays in the upward direction are larger than delays in the downward direction, reflecting the non-uniformity of delays depending on where the spike occurred (horizontal gradients in Figs 3, 4). Delay ratios in the spike S1 for evoked (1.53 +/− 0.29) and spontaneous activity (1.97 +/− 0.51) were significantly larger than for shuffled data (1.12 +/− 0.06), M = 0.41, 95% CI [0.24–0.62] and M = 0.85, 95% CI [0.54–1.24] respectively (Fig. 5E). Similar results were obtained for the other S1 where delay ratios in evoked (1.71 +/− 0.33) and spontaneous activity (2.23 +/− 0.47) both where larger than for shuffled data (1.11 +/− 0.04); M = 0.68, 95% CI [0.54–1.04] and M = 1.12, 95% CI [0.71–1.40] respectively (Fig. 5F).

The second difference with real data was that spike delays based on shuffled data showed systematic delays in the downward direction: for spikes in each layer, the delays in the downward direction followed spike rates, whereas in real data downward delays were more uniform (cf. Figs 3A,D, 4A). Together, these findings show that the larger delays observed in real data after spikes in deep layers do not result from differences in spike rates and that while spike rates co-determine delay patterns, they do not account for the observed pattern of upward propagation.

For spike-to-spike delays between hemispheres, shuffled data showed similar delays within the spike S1 and the other S1, and the delays for spikes in the other S1 were markedly shorter (<5 ms) than for real data (7–19 ms, Fig. 3B,C). This indicates that for real data the observed delays in the other hemisphere reflect activity propagation, not simply spike-frequencies.

### Propagation velocities

**Figure 6:**
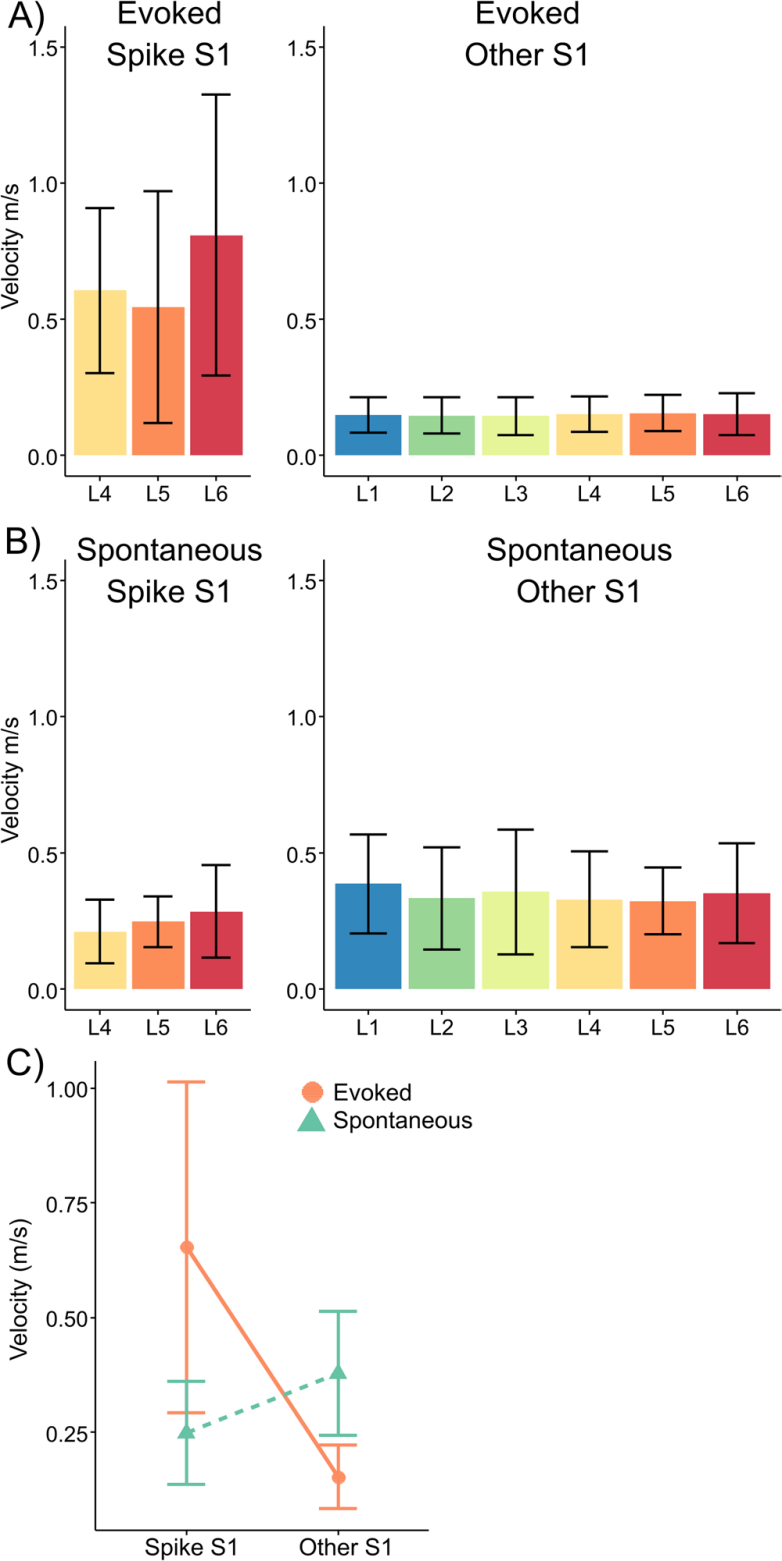
Propagation velocities. (**A**) Average velocities of upward propagation for evoked activity in the spike S1 (left) and in the other S1. (**B**) Upward propagation velocities for spontaneous activity. (**C**) Average and 95%confidence intervals for velocities relative to spikes in L4-L6 in evoked and spontaneous data, for the S1 where the spike occurred and the other S1. Error bar represent 95% confidence intervals.

To further investigate propagation velocities in evoked and spontaneous activity we modelled spike-to-spike delays as a linear function of cortical depth, where the estimated slope reflects the propagation velocity of population activity across cortical depth (m/s). In stimulus-evoked data, average velocity ranged between 0.51 and 0.84 m/s in the spike S1, and between 0.15 and 0.16 m/s in the other S1 (Fig. 6A). In spontaneous data, average velocity ranged between 0.21 and 0.29 m/s in the spike S1 and between 0.32 and 0.39 m/s in the other S1 (Fig. 6B). Average propagation velocities were subjected to a linear mixed effects model with Hemisphere (spike S1, other S1) and Activity (evoked, spontaneous) as fixed effects, and Animals as a random effect. This showed a statistically significant effect of Hemisphere *F*(1,15) = 5.9, *p* = 0.03 and a Hemisphere by Activity interaction *F*(1,15) = 17.1, *p* < 0.001 (Fig. 6C). Post-hoc tests using Tukey’s honest significant difference (HSD) correction showed that the interaction was driven by increased velocities for evoked activity in the spike S1. These were significantly higher than velocities for evoked activity in the other S1 (*p* < 0.001), than velocities for spontaneous activity in the spike S1 (*p* < 0.001) and marginally higher than velocities for spontaneous activity in the other S1 (*p* = 0.05). No other differences reached statistical significance (*p* < 0.05). These results suggest that evoked activity in the spike S1 (cS1 or iS1) propagates faster than evoked activity in the other S1, and generally faster than spontaneous activity.

### Propagation across cortical layers

We next asked whether the upward propagation reflected a step-wise process across layers, or rather a constant propagation through cortical tissue. To compare whether cortical layers or cortical depth best explained the upward delays we used the fact that the recording electrodes were unevenly spaced in depth. We used Bayesian model comparisons between delays modeled as a function of recording depth or as a function of layers (equally spaced distances). The evidence in favor of the layer model was quantified as a Bayes factor, see Methods^23,24^.

The results showed evidence in favor of a layer-like upward propagation, that is, Bayes factor values (BF-values) larger than 1 (Tables 1, 2); none of the model comparisons showed evidence in favor of the linear model (i.e. no BF < 1). For propagation in the spike S1 half of the B-values showed positive evidence for the layer model (i.e. BF > 3^23,25^), the other ones were smaller but still favored the layer model over the depth model. Lower BF-values were found particularly for delays following stimulus-evoked spikes in L4, reflecting the fact that with few data points, it is more difficult to distinguish the two model fits (e.g. after a L4 spike only delays in L3, L2 and L1 can be used to model the upward propagation). For upward propagation in the S1 opposite of the spike S1, positive evidence for the layer model was obtained in evoked activity while BF-values for spontaneous activity were smaller, but still favored the layer model.

**Table 1:** Bayes factors, spike S1.

Spike layer	Evoked activity	Spontaneous activity
L4	2.2	2.5
L5	2.7	3.3
L6	6.7	3.1

**Table 2:** Bayes factors, other S1.

Spike layer	Evoked activity	Spontaneous activity
L1	6.6	1.3
L2	6.4	1.7
L3	6.1	1.7
L4	5.7	2.0
L5	6.4	1.9
L6	6.3	1.8

In sum, the model comparisons suggest that the observed spike-to-spike delays are better understood as step-wise propagations over cortical layers than as a wave-like propagation across space.

## Discussion

We analyzed relative spike timings across cortical layers in bilateral S1 and found systematic and similar delay patterns in evoked and spontaneous activity that are in line with a predominant propagation of activity from infragranular to higher layers. In addition, we observed that relative to spikes in one S1, subsequent spikes in the other S1 are systematically delayed. The shortest interhemispheric delays occurred for infragranular layers and they increased with cortical layer, again consistent with a general upward propagation of spiking activity. These findings confirm a predominant upward propagation of activity in stimulus evoked data, and are in line with previous findings showing that inter-areal functional connectivity predominantly targets deeper layers (L4, L5, L6)^13^. Moreover, the results indicate that upward propagation is best understood as a step-wise propagation of activity across functional layers.

Our results are, to the best of our knowledge, the first to show upward activity propagation across all six cortical layers in both evoked and spontaneous activity. Together with previous results from mouse, cat and monkey, the current findings in rat S1 strongly suggest a canonical pattern of upward activity propagation across cortical layers. Previous work in visual cortex of cats and ferrets has demonstrated that slow oscillatory activity initiates ‘up-states’ of activity that start in L5 and propagate upward to supragranular layers^8^. Using multi-unit activity recorded from auditory cortex in awake mice, it was also shown that in spontaneous activity ‘up-states’ start in infragranular layers and propagate upward, while evoked activity showed a propagation from L4, downward and upward^6^. Information theoretic analyses also confirmed a driving role for infragranular layers in slow oscillations that target supragranular layers and the thalamus^11^. Upward propagation in spontaneous activity has been shown to be driven by excitatory L5 neurons, as demonstrated *in vivo* in cats, rats and mice^9,10,26^ and *in vitro* in rats^12^.

The upward propagation is also in line with results from LFP-based directed functional connectivity analysis in macaque and rats^11,13,16,27,28^. By analyzing dependencies between LFP signals recorded at different cortical layers these studies showed more directed interactions going from lower to higher layers than the other way around. This predominant upward flow pattern co-exists with downward interactions, like the bi-directional L2/3 - L5 coupling observed in both functional and structural connectivity^3,13,16,27,29^. Predominant downward flows have, however, also been reported, in infero-temporal cortex of macaques, where they appear to depend on functional state^27,30^. Our spike-to-spike delay results showed no indication for systematic delays in the downward direction. This could either be because downward delays are very fast and uniform, or because delays in the downward direction are less consistent than delays in the upward direction. Further work using spike-to-spike delays in other species should establish whether known downward connectivity can also be identified using this method.

Although spike rates for each layer partly determine the observed spike-to-spike delays, we controlled for this effect by shuffling the spike labels. The analysis of shuffled data resulted in a qualitatively different pattern of delays than that observed in the original data (Fig. 5C,D). This showed that differences in spike rates cannot account for the observed delay patterns because differences between up- and downward delays are higher in real data than may be expected based on spike rates alone (Fig. 5E,F).

The measure of population activity does not distinguish between excitatory and inhibitory units and our results therefore reflect the accumulated effects of excitatory and inhibitory cells. Compared to excitatory circuits, inhibitory circuits show greater diversity across cortical areas^31^. Recently, the importance of inhibitory interneuron activity has been well-established across different cortical lamina^32–34^. A spike-to-spike delay analysis that separates excitatory and inhibitory units could very well establish whether excitatory and inhibitory unit activity is related to similar functional propagation patterns. In our data, the recording sampling rate (6250 Hz) unfortunately prohibited spike sorting. Such future work should also include recordings from neighboring columns to better account for possible horizontal contributions to the vertical delay patterns^7,31,35,36^.

The function of upward propagation may involve the integration of activity from granular and infragranular layers with the activity in supragranular layers. Supragranular layers show more precise orientation tuning in macaque^37^ and play an important role in summation across visual space^38,39^. In contrast to L5, L2/3 reflect sparse coding, as shown in mouse auditory cortex^6^. A similar increase of sparsity from infragranular to supragranular layers was apparent in our data (Figs 1, 5A), in line with greater selectivity of responses in higher layers. Activity in supragranular layers may thus reflect more selective, relatively local processing than activity in infragranular layers. Upward propagation of activity is one mechanism by which infragranular layers can drive and shape activity in supragranular layers. This role for upward propagation is supported by the finding that L2/3 activity does not spread to neighboring columns in the absence of L5 neurons^12^. Conversely, silencing activity in L2/3 does not abolish visual responses and selectivity in L5^40^, showing an asymmetric dependency between infra- and supragranular layers that has been confirmed using optogenetics^26^.

The good convergence between results from spiking activity and functional connectivity analyses, in multiple species, strongly suggests that upward propagation may be a canonical pattern of information flow across cortical layers. This would constitute the functional correlate of the well described dense vertical axonal inter-connections of barrel column neurons across the different layers^3^. This pattern is best viewed as a simplified functional description of the complex underlying ascending and descending, axonal and dendritic, connections between cortical layers.

The observed similarity of upward propagation in evoked and spontaneous activity appears at odds with the classical model where activity is relayed via thalamo-cortical projections to L4, and then to other cortical layers of cS1. This spread from L4 has been repeatedly demonstrated using onset latencies and peak latencies of spiking activity^4,6^, as well as current source density (CSD) analyses^15,19,41^. However, recent work showed that L4 and L5 neurons have the same onset latencies^7^. A similar pattern of onsets is present in our results too, the earliest peak latencies of L4 and L5 spiking activity preceded those of other cortical layers (Fig. 2), in line with known thalamo-cortical projections and excitatory connections between cortical layers^2,3,42,43^.

The apparent discrepancy between the spread of evoked activity as described in the classical model and the systematic upward propagation observed from spike-to-spike delays can be resolved by the fact that onset and peak latencies represent single time-points in an ongoing response, while spike-to-spike delays summarize timing relationships across the entire response. Our results suggest that the sequential activation of cortical layers after stimulation does not dominate the relative spike timings across post-stimulus time, but that it gives way to a pattern of upward propagation, much resembling that observed in spontaneous activity. This is supported by a recent study of spike sequences across cortical layers which showed that only the initial spike sequences after stimulation are led by L4 neurons, while the later ones are led by L5 neurons^44^. Onset and peak latency analyses may thus provide a picture of evoked activity that is more the exception than the norm. Because they take a larger time window of activity into account, spike-to-spike delays and functional connectivity analysis may offer a more general characterization of evoked activity patterns. Consistent with previously reported similarities between evoked and spontaneous activity^45,46^, spike-to-spike delays thus confirmed in new ways that both evoked and spontaneous activity are well-characterized by predominant upward information flows.

Spiking activity in iS1 started in infragranular layers and spread to progressively higher layers, preceding the more sparse activity in supragranular layers in a seemingly orderly progression (Fig. 1). The upward propagation was confirmed in the spike-to-spike delay analysis, and closely resembled previously observed spread of spontaneous activity^6^, as well as results from functional connectivity analysis of these data that suggested a pattern of predominant upward propagation in iS1^13^. The similarities between evoked and spontaneous activity appeared despite clear differences in propagation velocities and spike rates. Evoked activity propagated faster in the spike S1 than spontaneous activity (Fig. 6), and spike rates were much elevated in evoked activity. A faster propagation for evoked than spontaneous activity was previously observed in the horizontal direction in rat barrel cortex^10^.

Several mechanisms could account for activity propagation across cortical tissue, and the observed propagation velocities of between 0.15 and 0.84 m/s help to exclude some explanations. These velocities are too high for spike propagation by synaptic integration. The minimal synaptic integration time of about 2 ms exceeds the observed delays between adjacent layers. The apparent lack of an integration time that separates activity in one layer from another can be expected when analyzing population activity. The observed delays between hemispheres, however, can reflect the relay of activity from one S1 to the other through synaptic integration since they exceed synaptic integration times by a large amount. We can also exclude propagation via electric field effects as a possible mechanism, because the slowest propagation in our data (0.15 m/s) is still faster than the typical velocity of field effects (0.1 m/s^47^).

Another mechanism that could account for the spike-to-spike delays is propagation of spikes along axons and dendrites. Propagation velocities along axons can range between 0.1 and 100 m/s, depending on the axon’s thickness and myelination^48,49^. In rat barrel cortex, propagation velocities along dendrites have been reported of around 0.26 m/s that are well in line with the current results^50^. The higher velocities observed for evoked activity match those observed for axonal propagation^10^. However, propagation along axons and dendrites would result in a uniform propagation across space while our results showed that upward propagation was better modeled as a step-wise increase with layers than as a linear increase with cortical depth. Therefore propagation along axons or dendrites cannot fully explain our results. Rather, the activity spends a constant time at each layer, irrespective of its thickness and the upward propagation is best thought of as a step-wise propagation across layers. Given these considerations the most likely mechanism may be a phase-lagged synchronized spiking of population activity across layers, possibly through LFP fluctuations that maintain neurons highly depolarized^51,52^. But further research is needed to establish this mechanism, and to separate contributions from nearby columns to upward propagation from contributions of other cortical areas and the thalamus, which project to multiple layers in S1 and could contribute to the observed upward propagation of activity^3,7^. It seem unlikely, however, that parallel activation of cortical layers via thalamo-cortical input could fully account for our findings because thalamic activity is unlikely to be sustained over the entire evoked response in cS1 (0–60 ms), is not the main driver of activity in iS1, and is only one of several contributors to spontaneous activity in S1.

Upward propagation may act to integrate activity from other areas into local processing. In line with this, our current and previous findings indicate that S1-S1 interactions predominantly target the infragranular layers of S1 in the other hemisphere^13^. Relative to spikes in one S1, delays in the other S1 were shortest at infragranular layers, both for evoked and spontaneous activity. S1-S1 interactions have been previously demonstrated and in bilateral whisking may allow for inhibition of activity on one side while actively sensing with the other^21,53^. These interactions can go over direct callosal projections, but are likely to include multiple indirect cortical and subcortical pathways^14,17,21,22^.

Although our S1-S1 delay patterns partially reflect spike rates, the delay patterns are unlikely to be fully explained by them. First, in evoked data there are consistent small delays for L6 in the other hemisphere that are absent in shuffled data. Second, in contrast to shuffled data, real data showed non-uniform delays depending on spike layer with longer delays for spikes in e.g. L1 after a spike in L5 of the other hemisphere than after a spike in L3. This is in line with a targeting of deeper layers and subsequent upward propagation of activity. And lastly, using a functional connectivity measure based on temporal precedence that is independent of spike rates we previously showed that the main targets from layers in one hemisphere are the deeper layers in the other hemisphere^13^. The finding of a specific targeting of the deeper layers also agrees with known dense structural connections between the S1s that project to infragranular layers^14,17,54^.

Inter-areal connections have been shown to specifically, although not exclusively, target L5 and L1^29,54–58^, which are the starting and the end point of the upward propagation. Inter-areal interactions that target L5 can therefore be expected to have different effects on local processing than those targeting L1. Inter-areal interactions that target L5 may relay information for integration with local computations in L2/3 of the target area, and change the content of local processing. Our findings showed a specific targeting of infragranular layers in S1-S1 interactions^13,14,17^. The observed targeting of deep layers and the subsequent upward propagation are consistent with the idea that activity relayed from one S1 is integrated with the local processing of the other S1 via upward propagation to L2/3. In good agreement with this, feedback during the presentation of illusory Kanisza triangles selectively activates infragranular layers in V1 in human, and may account for how the content of unstimulated visual field locations is updated^59^.

Inter-areal interactions that target L1, by contrast, have been shown to have a modulatory effect. L1 consist mainly of a dense network of dendrites and relatively few neurons, but post-synaptic potentials in L1 can modulate activity in lower layers through direct inhibition and apical dendrites^3,60–64^. Inter-areal interactions that target L1 are thus well-positioned to modulate how new information from infragranular layers is integrated into local processes. L1 has been characterized as the cortical layer where bottom-up, feedback and top-down influence converge^60^. L1 activity has been shown to have a modulatory influence on L2/3 in sensory processing^65,66^. A recent study in human provided evidence for contextual feedback through L1 in V1^67^. While these works suggest distinct functional roles for interactions targeting L5 or L1, a direct comparison between the effects of these types of inter-areal interactions on local processing is missing and will require careful causal manipulations since inter-areal interactions can simultaneously target infra- and supragranular layers^56,68,69^.

## Methods

Data were previously recorded from six P28-30 Wistar rats under light isoflurane anesthesia (3% induction, 2.5% maintenance) and lowered to the minimal level preventing the hind limb withdrawal reflex (0.7–1.1%)^13^. Differential potentials were recorded with a custom-made amplifier (gain 5000) and converted to digital format (A/D converter DT3004, Data Translation, MA) at its maximal sampling rate of 6250 Hz. We recorded simultaneously in the two S1s using linear depth probes (NeuroNexus Technologies, Ann Arbor, MI, USA) inserted perpendicularly to the cortical surface with contact points at depths corresponding to each cortical layer. The uppermost electrode was positioned at pia, the other electrodes were spaced in depth based on histology as follows: 100 µm from pia (L1), 300 µm (L2), 500 µm (L3), 800 µm (L4), 1100 µm (L5b), and 1500 µm (L6). Correct depth positioning was verified both during recording and offline. The probes were painted with a fluorescent marker and after the recordings; tangential sections of the parietal cortices were cut and processed for cytochrome oxidase staining to reveal the barrel field organization. The Dye I-marked electrode positions were checked under the microscope by switching the fluorescence filter on and off. Histology showed all penetrations were in barrel hollows E1, E2, or E3, but not necessarily in the same hollow in the two S1s. Further details on recordings and histology can be found in Plomp *et al*.^13^.

Unilateral whisker stimulation consisted of alternating left or right whisker stimulation (n = 100 for each) delivered to all large whiskers simultaneously through a solenoid-based stimulator device with an long inter-stimulus interval of 9 s^18^. Stimulation consisted of one 500 µm back-and-forth deflection (1 ms rise time).

Ethics approval was obtained from the local ethics committee (Office Véterinaire Cantonal; no. 31.1.1007/3208/2); all procedures conformed to Swiss federal law and EU Directive 2010/63/EU on the protection of animals used for scientific purposes. Further details are described in^13^, which reports the functional connectivity results of evoked data.

### Spike-to-spike delays

Spiking activity was identified at each cortical layer by thresholding the negative amplitude of 500–3000 Hz bandpassed LFPs with a data-driven threshold of 5 standard deviations per channel^20^. Spiking activity detected between 0 and 5 ms after stimulation onset was excluded from the analyses to avoid the possibility of mechanical stimulator artifacts influencing the results.

For every detected spike at time t we determined the delay (t + Δt) with which the next spike occurred in each of the other layers, allowing for a maximal delay of 30 ms. We computed average spike-to-spike delays for each layer of the S1 where the spike occurred (spike S1) and for each layer of S1 in the other hemisphere (other S1), for both stimulus-evoked activity (5–60 ms after stimulation, 200 epochs per animal) and spontaneous activity (7 second epochs starting 2 seconds after stimulation, 200 epochs per animal). Average spike-to-spike delays were calculated within animal and averaged across animals (n = 7).

To control for the effect of spike frequencies per layer we randomly shuffled the 12 labels (contralateral L1-L6, ipsilateral L1-L6) across all spikes of evoked activity and recalculated the spike-to-spike delays (1000 repeated shuffles per animal).

To test against the null-hypothesis of no difference between conditions we used non-parametric bootstrapping (n = 5000) to determine whether the 95% confidence intervals of the difference overlapped with zero (no difference). This is a suitable approach for small sample sizes without assumptions about the distribution of the dependent variable.

### Propagation velocity

To estimate upward propagation velocity, linear models were fitted to the average delays as a function of cortical depth using a least-squares approach and data from each animal. Preliminary analysis showed that in evoked data the propagation velocities derived from delays relative to spikes in cS1 and iS1 were very similar (compare Fig. 3A–D). The spike delays relative to cS1 and iS1 spikes were thus averaged within animals before velocity estimation.

For velocities in the spike S1 only the delay data relative to spikes in L4-L6 were used because these have enough data points in the upward direction (3–5 points, respectively) to reasonably fit a linear model. For delays in S1 opposite of the spike, models were fit to the delays obtained relative to spikes at each of the six layers.

### Bayesian model comparisons

By comparing the fits of two competing linear models we determined whether the data show more evidence for an increase with depth or for a layer-like progression of activity. For fitting the layer model (M_1_) we used equally spaced depths (−0.1, −0.38, −0.66, −0.94, −1.22, −1.50 mm) while for fitting the depth model (M_2_) we used the un-equally spaced actual recording depths (−0.1, −0.25, −0.50, −0.75, −1.10, −1.50 mm). In the Bayesian statistical framework the evidence for a model given the data is the posterior probability and the degree to which the data favor one model (M_1_) over another (M_2_) can be expressed as the ratio of their posterior probabilities, called the Bayes factor^23,24,70^:

1 $${B}_{12}=\frac{\Pr ({M}_{1}|data)}{\Pr ({M}_{2}|data)}=\frac{\Pr (data|{M}_{1})}{\Pr (data|{M}_{2})}\times \frac{\Pr ({M}_{1})}{\Pr ({M}_{2})}$$

where the posterior probabilities are the product of the marginal likelihood and the prior probability of each model, as per Bayes’ theorem. The layer model (M_1_) and the depth model (M_2_) were assumed equally likely a priori and their summed prior probabilities set to 1. The resulting Bayes factor values (BF-values) indicate the relative amount of evidence in the data. BF-values can range between 0 and infinity; for B_12_ > 1 the evidence favors M_1,_ for B_12_ < 1 the evidence favors M_2_. BF-values over 3 are generally considered positive evidence for a model, conversely, B-values smaller than 1/3 indicate positive evidence for the alternative model^23,25^. BF-values around 1 indicate the data cannot distinguish between the two models.

The Bayes factor can be approximated using the Bayesian Information Criterion (BIC) values of the two competing models^70^:

2 $${B}_{12}=\exp (-\frac{BI{C}_{1}-BI{C}_{2}}{2})$$

This is convenient in practice and allows model comparisons that penalize for the number of parameters. We fitted the two competing linear models using least squares approximation, calculated their BIC values and derived the corresponding BF-value for each individual animal, and then averaged the BF values to avoid effects driven by outliers^71^. Model fitting and comparisons were done in the statistical software R (www.r-project.org).

## Electronic supplementary material

Supplementary information
