# Supplementary material for: Systematic population spike delays across cortical layers within and between primary sensory areas
Source: Sci Rep. 2017 Nov 10;7:15267. doi: 10.1038/s41598-017-15611-2 (PMC5681572; doi:10.1038/s41598-017-15611-2)
Supplement: Supplementary file 1 — Supplementary information [file 41598_2017_15611_MOESM1_ESM.doc]

SUPPLEMENTARY INFORMATION

**Systematic population spike delays across cortical layers within and between primary sensory areas**

Gijs Plomp

Christoph M. Michel

Charles Quairiaux

Supplementary Table S1. Mean and bootstrapped 95% CI of spike likelihood peak latencies (ms)

| Layer | cS1 | iS1 |
| --- | --- | --- |
| L1 | 8.43 (6.4-9.2) | 35.25 (30.59-37.79) |
| L2 | 8.35 (7.71-9.07) | 35.33 (32.96-37.33) |
| L3 | 8.37 (7.65-9.12) | 33.81 (28.83-37.2) |
| L4 | 7.68 (7.12-8.27) | 31.63 (26.91-35.28) |
| L5 | 7.71 (7.15-8.67) | 33.55 (29.87-36.24) |
| L6 | 7.92 (7.33-8.59) | 29.44 (26.43-30.93) |
